# Serum BCMA levels predict outcomes in MGUS and smoldering myeloma patients

**DOI:** 10.1038/s41408-021-00505-4

**Published:** 2021-06-24

**Authors:** A. Visram, C. Soof, S. V. Rajkumar, S. K. Kumar, S. Bujarski, T. M. Spektor, R. A. Kyle, J. R. Berenson, A. Dispenzieri

**Affiliations:** 1 Department of Medicine, University of Ottawa, Ottawa Hospital Research Institute, Ottawa, ON Canada; 2 Division of Hematology, Mayo Clinic, Rochester, MN USA; 3 Institute for Myeloma & Bone Cancer Research, West Hollywood, CA California, USA; 4 OncoTracker, West Hollywood, CA California, USA; 5 Oncotherapeutics, West Hollywood, CA California, USA; 6 Berenson Cancer Center, West Hollywood, CA California, USA

**Keywords:** Myeloma, Tumour immunology, Prognosis

## Abstract

Soluble BCMA (sBCMA) levels are elevated in monoclonal gammopathy of undetermined significance (MGUS) and smoldering multiple myeloma (SMM). However, the association between sBCMA levels and prognosis in MGUS and SMM has not been studied. We retrospectively analyzed sBCMA levels in stored samples from 99 MGUS and 184 SMM patients. Baseline sBCMA levels were significantly higher in MGUS and SMM patients progressing to MM during clinical follow up. When stratified according to the median baseline sBCMA level for each cohort, higher levels were associated with a shorter PFS for MGUS (HR 3.44 comparing sBCMA ≥77 vs <77 ng/mL [95% CI 2.07–5.73, *p* < 0.001] and SMM (HR 2.0 comparing sBCMA ≥128 vs <128 ng/mL, 95% 1.45–2.76, *p* < 0.001) patients. The effect of sBCMA on PFS was similar even after adjusting for the baseline MGUS or SMM risk stratification. We evaluated paired serum samples and found that sBCMA increased significantly in MGUS and SMM patients who eventually progressed to MM, whereas among MGUS non-progressors the sBCMA level remained stable. While our results require independent validation, they suggest that sBCMA may be a useful biomarker to identify MGUS and SMM patients at increased risk of progression to MM independent of the established risk models.

## Introduction

The B cell maturation antigen (BCMA), a member of the tumor necrosis factor superfamily, promotes survival and proliferation of plasma cells through signal transduction of the B cell activating factor (BAFF/BLys) and a proliferation inducing ligand [1–3]. The selective expression of BCMA on mature B cells and plasma cells (neoplastic cells, in particular) has made it an attractive therapeutic target for the management of plasma cells disorders, specifically multiple myeloma (MM) [4–6]. Gamma secretase, a ubiquitous intramembranous protease, sheds membrane bound BCMA and releases it into the serum [7]. Previous work has shown that while serum BCMA (sBCMA) level is present in healthy donors, the levels are higher among those with monoclonal gammopathy of undetermined significance (MGUS), smoldering multiple myeloma (SMM), and MM [8, 9]. Among patients with MM, increased sBCMA levels correlate with an increased neoplastic plasma cell burden in the bone marrow, as sBCMA levels decrease with response to therapy [9]. In patients with non-secretory MM, where disease monitoring is a challenge due to the absence of circulating monoclonal proteins, sBCMA levels correlated with disease activity based on positron emission tomography (PET) imaging and bone marrow findings [9]. However, the relative differences in sBCMA levels between MGUS and SMM patients, and the association between baseline levels of sBCMA and risk of progression to MM have not been assessed in these patient populations.

MGUS and SMM are precursor plasma cell disorders that are associated with an increased risk of progression to MM. In MGUS patients, the risk of progression to MM is ~1% per year [10]. In SMM patients, the risk of progression to MM is ~10% per year for the first 5 years post diagnosis, 3% per year for the subsequent 5 years, and 1% per year thereafter [11]. While there are a number of additional factors associated with an increased the risk of progression to MM from MGUS or SMM – including immunoparesis of normal immunoglobulins, PET findings, and genetic aberrations – the most widely used risk stratification models for MGUS and SMM in our clinical practice rely on the baseline serum free light chain ratio (sFLC), monoclonal protein (MCP) level, bone marrow plasma cell burden, and monoclonal protein isotype [12–17]. However, the accuracy of sFLC, MCP, and plasma cell quantification may be limited by a number of factors, which may then lead to suboptimal risk stratification. The sFLC may be abnormal in patients with chronic kidney disease or disorders associated with reactive plasmacytosis [18, 19]. Migration of paraproteins, particularly IgA paraproteins, in the beta region of the serum protein electrophoresis can lead to inaccurate MCP quantification [20, 21]. Finally, the accuracy of bone marrow assessments for plasma cell involvement may be limited due to hemodiluted or poor-quality aspirate samples, patchy neoplastic plasma cell bone marrow involvement, and significant inter-observer variability due to the subjective nature of the plasma cell quantification [22–24]. However, prior studies have shown that there is no correlation between sBCMA levels and renal function [9], suggesting that sBCMA may be an objective and useful marker even in patients with renal dysfunction. Therefore, the aim of this study was first to assess whether sBCMA levels differed between MGUS and SMM patients at baseline and over time. The second aim was to assess the utility of sBCMA in prognosticating the risk of progression to MM and survival in MGUS and SMM patients.

## Methods

Within our MGUS and SMM biorepository, we selected three cohorts of patients with paired samples for study: (1) those with baseline MGUS that progressed to MM; (2) those with baseline MGUS that did not progress to MM during follow up; and (3) those with baseline SMM that progressed to MM during follow up. The second sample was obtained prior to initiation of anti-plasma cell therapy for all patients. In order to increase the SMM sample size, we included a fourth cohort of SMM patients who did not progress to MM during follow up and had a baseline sample only. On further review of the available baseline diagnostic studies, 8 patients met the revised 2014 criteria for MM [25] and were excluded from the study. An enzyme-linked immunoabsorbent assay with a polyclonal anti-BCMA antibody from R&D Systems (Minneapolis, MN, USA; catalogue #DY193E) was used to measure the sBCMA levels, as previously published [8, 9]. Serum BCMA levels were presented as the mean of triplicate samples for each specimen. In the older samples, there was no gating of the MCP and no clinical sFLC; and therefore, sample permitting, a serum protein electrophoresis and sFLC were also performed on each sample. Electronic medical records were reviewed to assess baseline clinical data as well as details regarding progression to MM. This study was approved by the Mayo Clinic Institutional Review Board.

### Statistical analysis

Descriptive statistics were used to quantify baseline characteristics. Between group comparisons were made using the non-parametric Wilcoxon test. A Spearman rank correlation was used to assess the correlation between sBCMA levels and baseline bone marrow biopsy plasma cell burden, monoclonal protein level, difference in the involved and uninvolved FLC (dFLC), and quantitative immunoglobulin of the involved heavy chain. Time to event analyses was performed using the Kaplan–Meier method in order to assess the progression free survival (PFS) and overall survival (OS). PFS was defined as the time from first sBCMA collection until death or disease progression. Similarly, OS was defined as time from first sBCMA collection until death, and patients were censored at last follow up. and OS were calculated from the time of the first sBCMA collection date. The Cox proportional hazards test was used to assess the hazard ratio for progression or death based on baseline sBCMA level, adjusted for MGUS or SMM risk stage, as well as baseline serum creatinine. Given that this study included patients diagnosed prior to the routine availability of cytogenetics on bone marrow biopsies, the 2/20/20 SMM risk stratification (risk factors include MCP > 2 g/dL, FLC ratio >20, bone marrow plasmacytosis >20%) [14] was used to adjust for baseline risk of progression to MM instead of the revised International Myeloma Working Group SMM risk model [15]. The MGUS risk stratification was based on the presence of a MCP ≥ 1.5 g/dL, non-IgG MCP isotype, or an abnormal FLC ratio [26]. Missing indicators were used to account for missing baseline staging in the Cox proportional hazards analyses. A Wilcoxon matched pairs signed rank test was used to assess for statistically significant changes between the sBCMA levels from samples collected at two different timepoints. A receiver operating curve was used to determine sBCMA levels that predicted the risk of progression to MM during follow up. Cohen’s kappa statistic was used to assess the concordance between the categorical variables. Statistical analyses were performed using JMP Pro v14.1 (SAS Institute, Cary, NC). A two-sided *p* value <0.05 was considered statistically significant.

## Results

A total of 283 patients were identified. Given the wide range of diagnosis dates (1965–2016), the baseline testing at diagnosis varied due to changes in clinical practice and the availability of diagnostic tests. Among those with a diagnosis of MGUS (*n* = 99), 24 had no imaging to exclude osteolytic lesions, only 25 patients had a baseline bone marrow biopsy evaluation, and 20 did not have a baseline sFLC. Similarly, among the patients with a diagnosis of SMM (*n* = 184), 3 had no imaging to exclude osteolytic lesions at diagnosis, and 47 had no sFLC within 3 months of diagnosis. Given the time period of diagnosis, advanced imaging at SMM diagnosis was not the standard of care; therefore, only 20 (11%) of SMM patients included in this study had a PET-CT scan, whole-body CT scan or whole-body MRI at diagnosis. The baseline characteristics of the included study cohort are shown in Table 1. There was a modest correlation between the baseline bone marrow plasma cell burden and sBCMA level (Spearman’s *ρ* = 0.631, *p* < 0.001) among 82 MGUS and SMM patients with both a diagnostic bone marrow biopsy and sBCMA tested on a serum sample collected within 1 month of diagnosis.). Furthermore, no correlation between the baseline sBCMA level and serum creatinine was observed in the 127 MGUS and SMM patients with results available within 1 month of diagnosis (Spearman’s *ρ* = 0.111, *p* = 0.214). A poor correlation was detected between the sBCMA and quantitative immunoglobulin of the involved heavy chain within 1 month of diagnosis (*n* = 84 MGUS and SMM patients, Spearman’s *ρ* = 0.398, *p* < 0.001) and between the initial sBCMA level and MCP (Spearman’s *ρ* = 0.462, *p* < 0.001) or dFLC (Spearman’s *ρ* = 0.383, *p* < 0.001) collected at the same time-point. There was moderate concordance between high-risk score based on the Mayo 2018 “20/20/2” model (a score ≥2) and a high sBCMA (≥128 ng/mL) (Cohen’s kappa statistic 0.471, *p* < 0.001).

**Table 1:** Baseline characteristics of patients with a diagnosis of MGUS and SMM at first sample collection.

	MGUS (*n* = 99)	SMM (*n* = 184)
Female - *n*(%)	47 (47)	82 (45)
Median age - years (IQR)	68 (58–75)	64 (55–71)
Median percentage bone marrow plasma cells - *n*(IQR)	5 (3–9)^a^	20 (15–30)
Median hemoglobin - g/dL (IQR)	13.2 (12.3–14.3)	12.5 (11.4–13.7)
Median creatinine - mg/dL (IQR)	1.1 (0.9–1.4)	1.1 (0.9–1.3)
Median MCP - g/dL (IQR)	0.5 (0–1.4)	2.1 (1.4–2.9)
Median iFLC - mg/dL (IQR)	3.5 (2–11.9)	6.45 (2.8–20.3)
Evaluable for risk staging^b^ – *n*	79	137
0 risk factors - *n*(%)	19 (24)	43 (31)
1 risk factor - *n*(%)	24 (30)	36 (26)
2 risk factors - *n*(%)	34 (43)	41 (30)
3 risk factors - *n*(%)	2 (3)	17 (13)
Heavy chain isotype		
IgG - *n*(%)	52 (53)	138 (75)
IgA - *n*(%)	29 (29)	40 (22)
Other^c^ - *n*(%)	18 (18)	6 (3)
Light chain		
Kappa - *n*(%)	58 (59)	127 (69)
Lambda - *n*(%)	41 (41)	57 (31)
Median time between diagnosis and first sBCMA collection - months (IQR)	0 (0–6.5)	1.9 (0–7.6)
Median sBCMA at first sample collection (IQR)	77.3 (37.9–128.8)	128 (77.4–212)

^a^Baseline bone marrow biopsy results were available for 25 MGUS patients

^b^Risk factors for MGUS patients include a monoclonal protein ≥1.5 g/dL, an abnormal free light chain ratio, and a non-IgG monoclonal protein isotype [26]. Risk factors for SMM patients include a baseline bone marrow plasma cell percentage >20%, serum free light chain ratio >20, and baseline monoclonal protein >2 g/dL [14].

^c^Includes light chain, IgD, and IgM isotypes.

We found a weak negative correlation between sBCMA and uninvolved quantitative IgA levels (Spearman’s *ρ* = −0.466, *p* < 0.001). Polyclonal immunoglobulin levels were available at diagnosis for only 162 (88%) SMM patients, and 49 (49%) MGUS patients. Of these patients, 16 (33%) MGUS and 120 (74%) SMM patients had immunoparesis (defined as an uninvolved quantitative immunoglobulin below the lower limit of normal, and reference ranges for quantitative immunoglobulins at our institution are 61–356 mg/dL for IgA, 767–1590 mg/dL for IgG, and 37–286 mg/dL for IgM). There was poor concordance between the presence of immunoparesis and elevated sBCMA (≥128 ng/mL for SMM patients, ≥77 for MGUS patients), (Cohen’s kappa coefficient = 0.226, *p* < 0.001).

**Fig. 1:**
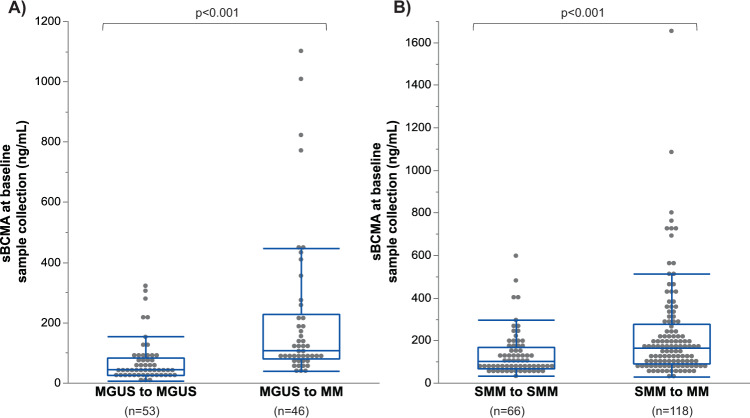
The value of the first sBCMA sample is shown in patients who progress to active MM during follow up, compared to those patients who do not progress. **A** Demonstrates that in MGUS patients, the median baseline sBCMA in patients that did not progress to MM during follow up was 43.7 (IQR 25.0–82.3) ng/mL, compared to 106.5 (IQR 78.9–227.3) ng/mL in patients that did progress to MM. **B** Demonstrates that in SMM patients, the median baseline sBCMA in patients that did not progress to MM during follow up was 101.7 (IQR 67.4–165.5) ng/mL, compared to 162.7 (IQR 90.5–275.8) ng/mL in patients that did progress to MM.

The first sBCMA sample was collected at a median of 0 (IQR 0–6.5) months of diagnosis in MGUS patients, and 1.9 (IQR 0–7.6) months after diagnosis among SMM patients. The median sBCMA level at the first sample collection was lower in MGUS patients (77.3, [IQR 37.9–128.8] ng/mL than those with SMM (128.1, [IQR 77.4–212.0], *p* < 0.001). In MGUS patients, the baseline sBCMA sample was significantly higher among those who progressed to MM during follow up (median 106.5 [IQR 78.9–227.3] ng/mL) compared to patients who did not progress (median 43.7 [IQR 25–82.3] ng/mL), as shown in Fig. 1A. The baseline samples from the cohort of MGUS patients who progressed to MM were collected at a later point in the disease course compared to the MGUS patients who did not progress (median time from diagnosis 0.3 [IQR 0–61.1] vs 0 [IQR 0–0] months, *p* < 0.001). However, even when limiting the analysis to those MGUS patients who had a sample for BCMA measurement within 3 months of diagnosis the baseline BCMA levels were significantly higher among the progressors (*n* = 25, median sBCMA level 95.0 ng/mL) compared to non- progressors (*n* = 43, median sBCMA level 40.1 ng/mL, *p* < 0.001). Similarly, the baseline sBCMA in SMM patients was also significantly higher in those who progressed to MM during follow up (median 162.7 [IQR 90.5–511.8] ng/mL) compared to patients who did not progress (median 101.7 [IQR 67.4–165.5] ng/mL), as shown in Fig. 1B. There was no significant difference in the time between diagnosis and initial sample collection among SMM patients who did versus did not progress to MM (1.8 vs 2.2 months, respectively, *p* = 0.578).

### Serum BCMA predicts PFS and OS in MGUS and SMM patients

**Fig. 2:**
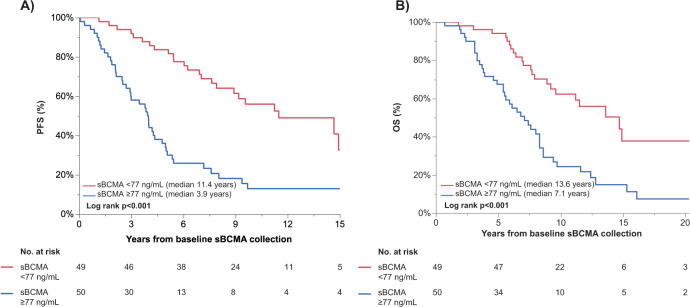
In MGUS patients (*n* = 99), the first sBCMA sample collected was used to stratify patients into two groups, those with sBCMA below versus equal to or above the median sBCMA at first sample collection. The **A** PFS, and **B** OS are shown, stratified by the median sBCMA values at first collection.

**Fig. 3:**
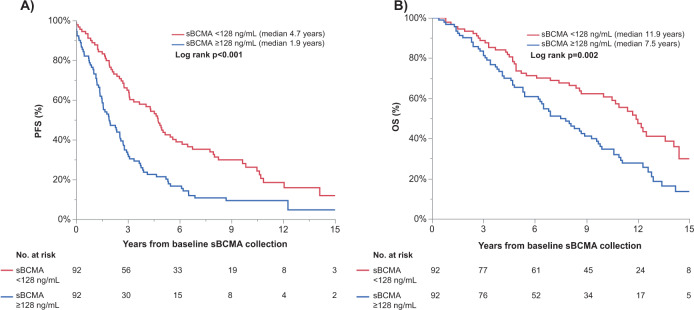
In SMM patients (*n* = 184), the first sBCMA sample collected was used to stratify patients into two groups, those with sBCMA below versus above the median sBCMA at first sample collection. The **A** PFS, and **B** OS are shown, stratified by the median sBCMA values at first collection.

Given that there are no established sBCMA cut-offs associated with an increased risk of progression to MM in MGUS or SMM patients, time to event analyses were conducted by stratifying patients into groups based on whether the first sBCMA sample was above or equal to the median sBCMA level for the cohort (as shown in Figs. 2 and 3). Thus, the median sBCMA cut-offs used were 77 ng/mL for MGUS patients and 128 ng/mL for SMM patients.

In MGUS patients, the PFS was significantly longer in patients with a baseline sBCMA below the median at 11.5 vs. 3.9 years (*p* < 0.001). The PFS hazard ratio for a baseline sBCMA ≥77 ng/mL was 3.4 (95% CI 2.1–5.7, *p* < 0.001); the results were no different when restricting to the subset of MGUS patients with sBCMA tested on a sample collected within 1 month of diagnosis (*n* = 68). After adjusting for baseline MGUS risk stratification [26], the PFS risk remained significant, though the effect was attenuated slightly (HR 2.6, 95% CI 1.5–4.6, *p* < 0.001), as shown in Table 2. In a univariable analysis, the addition of the baseline serum creatinine did not significantly affect the Cox proportional hazards model for PFS. The median OS was 8.6 (95% CI 7.6–11.5) years for the MGUS patient cohort. The risk of death, stratified by the sBCMA ≥77 versus <77 ng/mL, for the entire MGUS cohort and the cohort tested within 1 month of diagnosis was 2.7 (95% 1.6–4.6, *p* < 0.001) and 3.0 (95% CI 1.5–5.8, *p* = 0.002), respectively. The hazard of death was 2.3 higher (HR 2.3, 95% 1.2–4.1, *p* = 0.007) in MGUS patients with a baseline sBCMA ≥77 ng/mL, even after adjusting for the MGUS risk score. In total, 41 patients with MGUS died without progression to multiple myeloma.

**Table 2:** Uni- and multivariable Cox proportional hazards analyses for progression-free survival.

	Univariable analysis			Multivariable analysis		
	HR	95% CI	*p* value	HR	95% CI	*p* value
SMM patients (*n* = 184)						
Baseline FLCr >20 vs ≤20^a^	1.92	1.27–2.90	0.002	1.57	1.03–2.40	0.035
Baseline MCP > 2 vs ≤2 mg/dL	1.6	1.16–2.20	0.005	1.37	0.96–1.95	0.084
Baseline BM PC > 20% vs ≤20%	1.78	1.28–2.46	0.001	1.49	1.01–2.19	0.046
sBCMA at sample 1 ≥ 128 vs <128 ng/mL	2.00	1.45–2.76	<0.001	1.46	1.01–2.11	0.042
MGUS patients (*n* = 99)						
Baseline non-IgG vs IgG isotype	1.03	0.64–1.67	0.901	1.06	0.63–1.80	0.823
Baseline MCP ≥ 1.5 vs <1.5 g/dL	3.73	2.19–6.38	<0.001	2.43	1.32–4.47	0.004
Baseline abnormal FLCr^a^	2.42	1.34–4.36	0.003	1.85	1.01–3.40	0.046
sBCMA sample ≥77 vs <77 ng/mL	3.44	2.07–5.73	<0.001	2.59	1.48–4.55	<0.001

*MCP* monoclonal protein, *BM PC* bone marrow plasma cells.

^a^A missing indicator was used to account for missing data.

For SMM patients with BCMA values above or equal to the median (sBCMA ≥ 128 ng/dL), the median PFS was significantly shorter at 1.9 vs 4.7 years (HR 2.0, 95% CI 1. 5-2.8, *p* < 0.001). Even after adjusting for SMM risk incorporating MCP, FLC, and bone marrow plasmacytosis [14], the PFS risk was 1.5 (95% Cl 1.2–2.1, *p* = 0.042), as shown in Table 2. The median OS was 9.7 (95% CI 8–11) years for the SMM patient cohort. High BCMA also was a risk factor for OS even after adjusting for the baseline SMM risk score (HR 1.7, 95% CI 1.1–2.5, *p* = 0.015) as well as SMM risk score and serum creatinine (HR 1.6, 95% CI 1.1–2.3, *p* = 0.010). In contrast, when the Cox proportional hazards modeling was restricted to the 69 SMM patients with sBCMA levels from a sample collected within 1 month of diagnosis, baseline sBCMA was no longer a significant risk factor for PFS (HR 1.6, 95% CI 1.0–2.6, *p* = 0.061) and OS (HR 1.0, 95% CI 0.6–1.7, *p* = 0.919).

### Progression to MM is associated with an increase in sBCMA levels

**Fig. 4:**
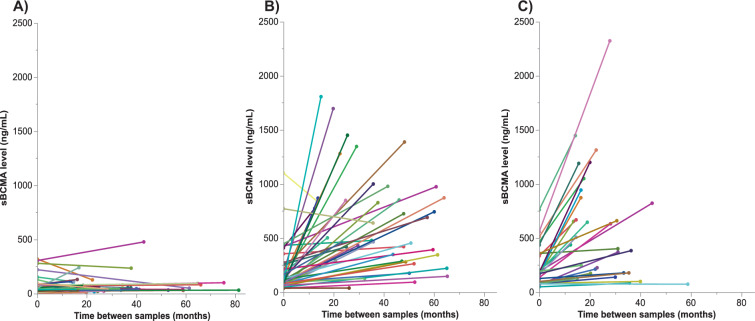
Demonstration of the change in sBCMA level between sample collection 1 and 2 in paired patient samples, accounting for the variability in the time between sample collection. The change in sBCMA is shown for patients with **A** MGUS who did not progress to MM (*n* = 49), **B** MGUS patients who did progress to MM (*n* = 42), and **C** SMM patients who progressed to MM (*n* = 29).

In order to assess whether sBCMA levels changed over time, paired samples were tested at two time-points in patients with MGUS (*n* = 42) and SMM (*n* = 29) patients that progressed to MM during follow up, as well as MGUS patients that did not progress to MM during follow up (*n* = 49; Table 3). The time between samples was not consistent between patients, and the median time between sBCMA samples 1 and 2 was 27 (IQR 16.5–42.6) months. The second sBCMA sample was collected within 30 days of progression to MM in 25 (86%) of SMM patients and in 40 (95%) of MGUS patients. In MGUS patients without progression, there was no significant difference in sBCMA over time (*p* = 0.184). In patients that progressed to MM, the median sBCMA increased by 312 (IQR 150–775, *p* < 0.001) ng/mL or 2.7 fold (IQR 0.9–7.4) for MGUS patients and by 303 (IQR 58–657, *p* < 0.001) ng/mL or 1.3 fold (IQR 0.7–2.9) among SMM patients. There was no significant difference in the median change in sBCMA in MGUS versus SMM patients who progressed to MM (*p* = 0.494), as demonstrated visually in Fig. 4.

**Table 3:** Changes in laboratory parameters in MGUS and SMM patients over time.

	MGUS to MGUS (*n* = 49)	MGUS to MM (*n* = 42)	SMM to MM (*n* = 29)	*p* value
Sample 1				
Months between diagnosis and collection	0 (0, 0)	0.3 (0–61)	0.7 (0, 3.15)	<0.001
MCP - g/dL	0 (0, 0.7)	1.1 (0, 1.8)	2.3 (1.8, 2.75)	<0.001
iFLC, - mg/dL	2.4 (1.8, 4.5)	4.6 (2.1, 11.9)	6.3 (2.4, 18.6)	0.025
dFLC - mg/dL	0.8 (0.4, 2.7)	4.4 (1.5, 12.6)	6.0 (3.2, 18.3)	<0.001
sBCMA - ng/mL	41.9 (24.1, 79.6)	106.5 (78.9, 227.3)	174.9 (84.3, 347.1)	<0.001
Months between collection of samples 1 and 2	20.8 (15.8, 37.8)	36.7 (24.4, 51.1)	22.1 (16.0, 32.2)	0.001
Absolute change over time				
MCP - g/dL	0 (0, 0)	2.1 (0.9, 2.9)	1.1 (0.7, 1.7)	<0.001
iFLC, - mg/dL	−0.2 (−0.6, 0.6)	18.4 (3.1, 71.3)	2.3 (0.3, 24.5)	<0.001
dFLC - mg/dL	−0.05 (−0.39, 0.56)	15.8 (4.1, 73.0)	2.1 (0.4, 24.5)	<0.001
sBCMA - ng/mL	2.7 (−9.4, 18.1)	311.6 (150.1, 774.5)	302.8 (57.7, 656.5)	<0.001
Fold change over time				
MCP - g/dL	0 (−0.3, 2.8)	1.8 (0.8, 599)	0.3 (0.6)	<0.001
iFLC, - mg/dL	−0.1 (−0.2, 0.3)	4.2 (0.8, 7.4)	0.6 (0.1, 2.5)	<0.001
dFLC - mg/dL	−0.01 (−0.5, 0.8)	4.9 (1.2, 13.8)	0.7 (0.1, 3.3)	<0.001
sBCMA - ng/mL	0.1 (−0.2, 0.5)	2.7 (0.9, 7.4)	1.2 (0.7, 2.9)	<0.001

Data are represented as median (IQR).

## Discussion

In this study, we showed that baseline sBCMA levels are significantly higher in SMM patients compared to MGUS patients. Furthermore, within the MGUS and SMM patient cohorts, the initial sBCMA levels were significantly higher in MGUS and SMM patients who eventually progressed to MM during follow up compared to patients that did not progress. We used the median sBCMA levels to stratify the MGUS and SMM cohorts into two groups and found that patients with a baseline sBCMA above the respective medians had significantly worse PFS and OS. Though the biological mechanisms to explain this association have not been elucidated, it has been hypothesized that malignant transformation in SMM and MGUS patients occurs due to both the clonal evolution of neoplastic plasma cells and alterations in the normal bone marrow microenvironment [27]. In murine MM models, sBCMA has been shown to sequester circulating BAFF, thereby decreasing the stimulation and quantity of normal B-cells and reducing the normal polyclonal antibody levels [28, 29]. Therefore, it is possible that sBCMA may lead to immune dysregulation in precursor plasma cell conditions, thereby increasing the risk of disease progression as well as infection. While our study showed only a moderate inverse correlation between sBCMA and quantitative IgA levels in IgG MGUS and SMM patients, we hypothesize that the degree of immunoparesis may be more significant at higher sBCMA levels.

In SMM patients, accurate prediction of the risk of progression to MM is particularly important given that currently there is clinical equipoise regarding the optimal management of patients with SMM. The conventional management has been to closely monitor patients for signs or symptoms to suggest evolution to symptomatic MM. However, given that early therapeutic intervention in high-risk SMM patients has been shown to decrease the risk of disease progression [30, 31], treating these patients may be beneficial. The use of aggressive regimens for a curative intent is being studied in the ASCENT (ClinicalTrials.gov NCT03289299) and GEM-CESAR (ClinicalTrials.gov CT02415413) trials. While BCMA-based therapies have not yet been studied in this context, given the promising efficacy of BCMA-based immune therapies in highly refractory MM populations [6, 32, 33], it is possible that they may be studied to treat high-risk SMM patients in the future. Identifying the subset of SMM patients that would benefit from aggressive treatment approaches, in order to justify their potential risks and toxicities is essential. This requires optimization of existing prognostication models. In our study, in both the MGUS and SMM cohorts, an elevated sBCMA level (above the median for the respective cohort) was associated with an increased risk of progression to active MM or death even after adjusting for the baseline MGUS or SMM risk stratification. This suggests that for both MGUS and SMM patients, the baseline sBCMA level has added prognostic value in addition to risk scores that are routinely used in current clinical practise [14, 26]. The revised IMWG SMM risk stratification, which incorporates cytogenetics and a more defined classification of laboratory and pathology tumor measurements, still has a positive predictive value of ≤50% for SMM with a risk score between 0 and 8 [15]. Therefore, additional markers such as the sBCMA may improve the ability of existing risk models to predict patients at higher risk of malignant transformation.

Our study has some limitations. The MGUS and SMM patients were selected to allow a comparison of those that progressed compared to those that did not progress. Therefore, our patient cohorts were skewed to include more progressors than general MGUS and SMM patient populations, and this likely led to an overestimation of the median sBCMA levels within the respective cohorts. Therefore, if the sBCMA cutoffs used in this study are applied to a general MGUS or SMM population, they may be less sensitive at identifying disease progression. Therefore, the sBCMA cut-offs used in this study require independent validation. Due to the retrospective nature of the study, the changes in clinical recommendations over time, and limited availability of diagnostic tests for patients diagnosed prior to 2005, baseline advanced imaging and sFLC were not available for all patients. Therefore, it is possible that a subset of patients included in this study may have met the revised SLiM-CRAB criteria for active MM and should not be part of these study populations [25]. This may explain why the median sBCMA level in SMM patients in this study was 128 ng/mL, whereas a study using the same sBCMA assay has previously reported that SMM patients had a median sBCMA level of 88.9 ng/mL [9]. While the samples included in this study corresponded to patients diagnosed over a large time span (1965–2016), we do not think this would affect our results regarding the association between sBCMA and progression risk given that the standard of care during this time period was to monitor patients without therapy. However, due to significant advancements in myeloma therapy during this time period, patients progressing to MM in recent years may have an improved overall survival given the availability of novel agents such as proteasome inhibitors, immunomodulatory drugs, monoclonal antibodies, and the routine use of autologous stem cell transplantation among eligible patients. Therefore, the time period of MM diagnosis and subsequent treatments likely confounds the association between sBCMA levels and survival.

In this study, we showed that sBCMA levels increase in SMM and MGUS patients that progress to MM. However, given the heterogeneity in the timing between paired samples, and the fact that second sBCMA was collected at MM progression in most patients, our study does not address whether changes in sBCMA levels have added prognostic ability compared with standard tumor markers such as MCPs and sFLCs. It would be beneficial to prospectively study the changes in sBCMA levels over time, to see if this biomarker could be used to detect evolution of MGUS/SMM to MM prior to the development of end organ damage. At present, due to the limited availability of the sBCMA test in clinical laboratories, it is unlikely to be incorporated into routine prognostic risk models or used for monitoring MGUS or SMM very soon. However, given that BCMA-based therapies have shown promising efficacy in MM patients, the availability of BCMA assays is likely to increase in the future.

In conclusion, our study shows sBCMA levels are increased in precursor conditions such as MGUS and SMM. More importantly, increased baseline sBCMA levels in MGUS and SMM patients were associated with an increased risk of progression and death. Though our results require independent validation in order to establish clinically relevant sBCMA cut-offs, these data suggest that sBCMA may be a useful biomarker to identify patients at increased risk of progression to MM.
